# Recovery of *Cryptococcus gattii* from an Infected Ventriculo-Peritoneal Shunt, Illinois, USA

**DOI:** 10.3201/eid2407.171754

**Published:** 2018-07

**Authors:** Donna Moritz, Alfredo J. Mena Lora, Bridget Blumer, Amanda T. Harrington

**Affiliations:** University of Illinois at Chicago, Chicago, Illinois, USA (D. Moritz, A.J. Mena Lora);; ACL Laboratories, Rosemont, Illinois, USA (B. Blumer);; Loyola University Medical Center, Maywood, Illinois, USA (A.T. Harrington)

**Keywords:** Cryptococcus gattii, cryptococcoma, ventriculo-peritoneal shunt, Illinois, United States, fungus, fungal infection, fungi

## Abstract

*Cryptococcus gattii* is a fungal pathogen endemic in tropical and subtropical regions. Isolated cases and outbreaks have been reported in areas of North America and Europe, expanding the distribution pattern beyond warmer regions. We describe a case of ventriculo-peritoneal shunt infection by *C. gattii* in an immunocompetent person in Illinois.

*Cryptococcus gattii* is a fungus found in soil and decaying organic materials (*1*–*3*). *C gattii* infections have been reported in tropical and subtropical regions worldwide. In the United States, *C. gattii* human infection is rare; <300 cases have been documented, of which 169 were reported to the Centers for Disease Control and Prevention (CDC) during 2005–2013 (*4*). Most cases were reported in southern California before a rise in cases occurred after 1999 in the Pacific Northwest (*2*,*5*). We report a case of ventricular shunt infection by *C. gattii* in an immunocompetent person in Illinois.

A 40-year-old man from Lake County, Illinois, with no known medical problems was admitted in October 2015 for evaluation of hydrocephalus. The patient reported 4 months of throbbing frontal headaches, nausea, and vomiting. Progressive confusion, altered memory, intermittent gait, and balance disturbances also were present. No visual changes, fevers, chills, or seizures were reported. The patient had no travel outside of Illinois and no ill contacts. Computed tomography (CT) scan of the brain demonstrated hydrocephalus, which was concerning because it indicated possible abnormalities in the flow of cerebrospinal fluid (CSF). A right frontal ventriculostomy catheter was placed. Repeat CT imaging of the head showed a possible mass within the right cerebellar hemisphere and surrounding vasogenic edema. To determine whether an infectious pathogen was the cause, we performed a workup that included HIV screening, *Echinococcus* serologic testing, interferon gamma release assay, cysticercosis serologic testing, and 3 CSF cultures; all results were negative. Results of a complete blood count with differential and comprehensive metabolic panel were unremarkable. The patient was not receiving immunosuppressive therapy nor had any other known risk factors associated with immunosuppression. A right ventriculo-peritoneal (VP) shunt with a programmable valve was placed, and the patient was discharged to home in stable condition. 

One month later, the patient was readmitted with recurring symptoms. CT imaging of the head showed stable ventricular size. Contrast-enhanced magnetic resonance (MR) of the brain showed abnormalities above the tentorium, possibly representing a cystic mass obstructing the foramen of Monroe bilaterally, with pronounced distention of both lateral ventricles. CSF studies showed a leukocyte count of 3/μL (reference range 0–5/μL) with lymphocytic predominance (93% [reference range 40%–80%]) and protein level of 42 mg/dL (reference range 15–45 mg/dL). We observed large round yeasts on Gram stain of CSF. The VP shunt was externalized. Cryptococcal antigen (Immy; Norman, OK, USA) was positive in the CSF (1:160 titer). Cultures from the CSF grew yeast that we identified as *Cryptococcus neoformans* by using matrix-assisted laser desorption/ionization time-of-flight mass spectrometry (Vitek MS IVD Database version 2; bioMérieux, Durham, NC, USA). We then subcultured the organism to CGB Agar (L-canavanine, glycine, bromothymol blue; Hardy Diagnostics, Santa Monica, CA) to differentiate *C. neoformans* from *C. gattii*. The organism, which produced blue coloration on CGB agar, was determined to be *C. gattii* and was confirmed as *C. gattii* molecular biotype VGI by multilocus sequence typing performed at CDC (Atlanta, Georgia, USA). Induction with amphotericin B and flucytosine was given for 14 days and high-dose fluconazole (800 mg/d) was subsequently given as consolidation therapy for 8 weeks. The dose was then decreased (to 200 mg/d) for maintenance therapy. The patient was lost to follow-up after his first outpatient clinic visit.

We postulate that our patient likely had a cryptococcoma with a low organism burden on initial presentation. We found no cases of VP shunt infection attributable to *C. gattii* in the literature. Only 10 cases of VP shunt infections attributable to *C. neoformans* have been reported; the time from shunt placement to symptom onset ranged from 10 days to 20 years (*6*,*7*). Six of 10 cases resulted from shunt placement in persons previously infected (*6*,*8*). The patient we report had onset of symptoms 4 weeks after VP shunt placement, likely reflecting an underlying infection before VP shunt placement.

Only 4 isolates of *C. gattii* have been identified from the Midwest region of the United States; these isolates were identified as VGI and VGIII types (S. Lockhart, CDC, pers. comm., 2017 Jul 18) (*9*). A recent study demonstrated that a large subset of isolates from throughout the United States were VGI, including a cluster of isolates with a single multilocus sequence type originating in the southeastern United States (*10*). The isolate in this case was identified as molecular type VGI and by multilocus sequence typing was shown to have the same sequence type as isolates from patients in Florida and Georgia and isolates from the environment in Washington.

Infections attributable to *C. gattii* are not confined to tropical and subtropical regions. The case we describe serves to extend the known range of this organism to include Illinois. Infections might be missed, given that many laboratories do not routinely differentiate *C. gattii* from *C. neoformans*. Mortality rates can range from 13% to 33% (*4*). Thus, clinicians and laboratorians must have increased awareness of this emerging infectious disease.
